# Supplementary material for: Exploring the Relationship between the Gut Mucosal Virome and Colorectal Cancer: Characteristics and Correlations
Source: Cancers (Basel). 2023 Jul 9;15(14):3555. doi: 10.3390/cancers15143555 (PMC10376985; doi:10.3390/cancers15143555)
Supplement: Supplementary file 1 [file cancers-15-03555-s001.zip › cancers-2407964-supplementary.pdf]

Supplementary figures and table

Figure S1. The contribution of viral taxa to the differences between the gut virome among the groups.

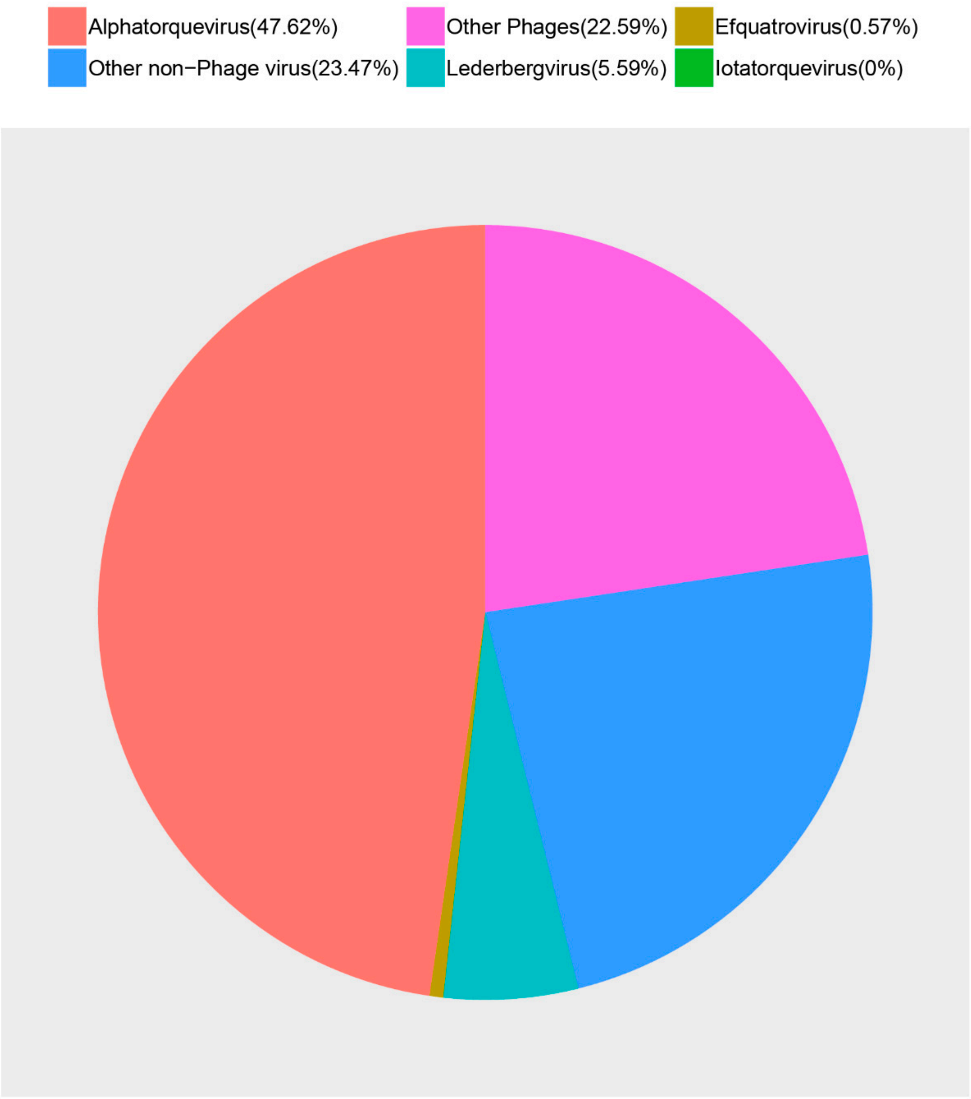

Figure S2. The viral composition of samples from HCs, CRC-A and CRC at different levels. Different viral taxa among HCs, CRC-A and CRC at the family (a), genus (b) and species levels (c).

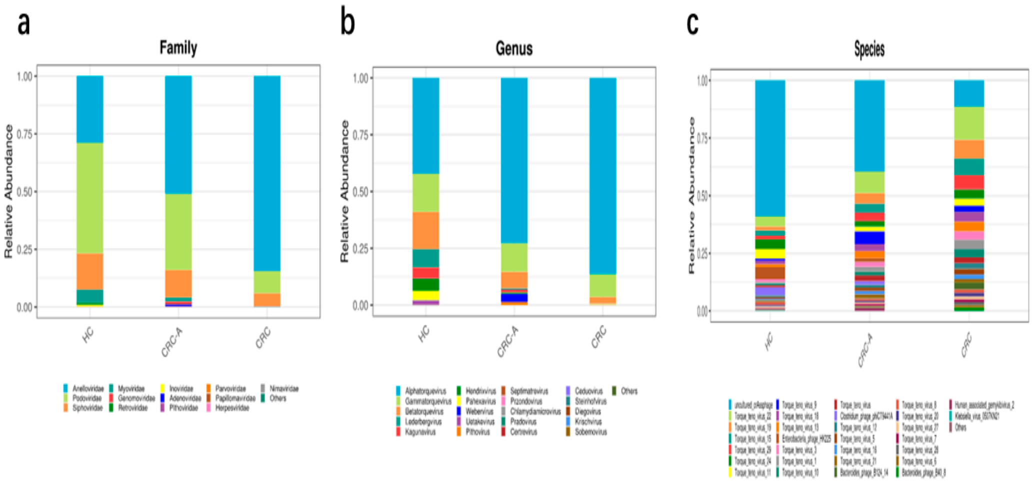

Figure S3. Relative abundance of viral species between early and late stage of CRC.

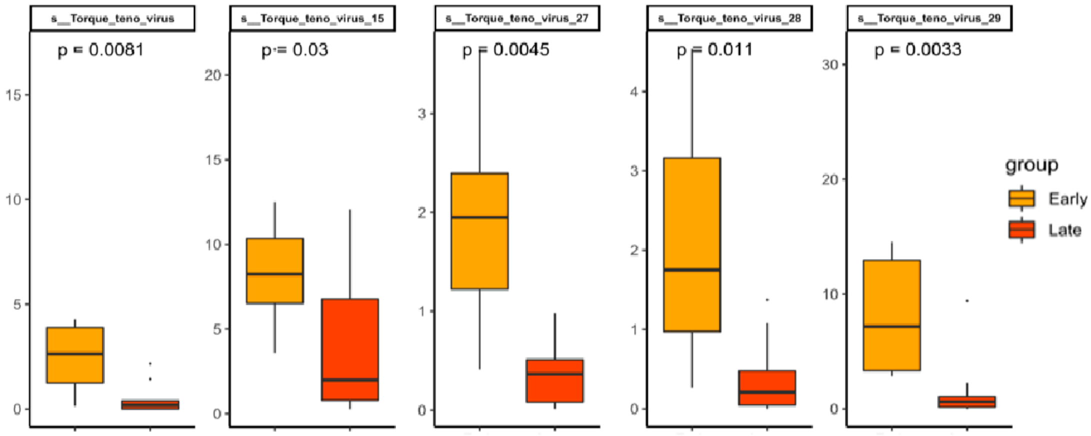

Table S1. Positive rate of Hapillomavirus, Herpesvirus, Polyomavirus and Hepatitis virus in samples.

	HC(%)	CRC-A(%)	CRC(%)
Papillomavirus-6	0/15	1/18	0/18
Herpesvirus-2	0/15	1/18	1/18
Herpesvirus-4	0/15	0/18	1/18
Polyomavirus-1	0/15	0/18	1/18
Hepatitis virus	0/15	0/18	0/18
